# A Prospective Cross‐Sectional Cohort Study on Factors Associations With Right Heart Thrombi and Pulmonary Embolism

**DOI:** 10.1002/hsr2.70825

**Published:** 2025-05-07

**Authors:** Wendlassida Martin Nacanabo, Taryètba André Arthur Seghda, Djième Claudine Dah, Mureille Loya, Lamoudi Prisca Thiombiano, Wendlassida Léa Françoise Sawadogo, Ella Hatoula Lengani, Anna Tall/Thiam, Nobila Valentin Yameogo, André Koudnoaga Samadoulougou, Partice Zabsonre

**Affiliations:** ^1^ Cardiology Department Bogodogo University Hospital Center Ouagadougou Burkina Faso; ^2^ Cardiology Department Yalgado Ouedraogo University Hospital Center Ouagadougou Burkina Faso

**Keywords:** Burkina Faso, Ouagadougou, right atrium, right ventricle, survival, thrombosis

## Abstract

**Introduction/Objective:**

Floating thrombus of the right heart chambers is an uncommon diagnosis and is systematically associated with pulmonary embolism. The aim of this study is to describe the management and evolution of right intracavitary thrombi (TIC) in pulmonary embolism.

**Materials and Method:**

This was a prospective cohort that ran for 76 months from March 5, 2017 to July 31, 2023. Patients diagnosed with pulmonary embolism were included. Two population groups were established according to the presence or absence of right intracavitary thrombus on Doppler echocardiography (intracavitary thrombi+ vs. intracavitary thrombi−) permitted to compare the risks factors. Thromboembolic risk factors, including the patient's background and clinical, paraclinical, therapeutic and evolutionary aspects, were reported. A significance level of *p* < 0.05 was used for data analysis.

**Results:**

The prevalence of emboli associated with intracavitary thrombi was 2.98%. Arterial hypotension, right ventricular dilatation, and deaths were significantly associated with right chamber thrombi with *p* = 0.0051 (OR: 4.14; IC 95%: 1.53–11.2); 0.0015 (OR: 4.4; IC 95%: 1.76–10.9); 0.00 (OR: 9.21; IC 95%: 3.69–23.3); 0.0313 (OR: 3.16; IC 95%: 1.10–9.02), respectively. Thrombolysis was performed in 70.58% of patients in the intracavitary thrombi+ group, compared with 11.93% in the intracavitary thrombi− group. The presence of intracavitary thrombi+ was associated with a high mortality rate of 35.29% compared with 9.82% in the intracavitary thrombi− group.

**Conclusion:**

The discovery of a thrombus of the right heart, although rare, is not exceptional. Their management is the subject of controversy between learned societies. Thrombolysis is the only therapeutic option in this context.

## 1 Introduction

Floating thrombus of the right heart chambers is an uncommon diagnosis and is systematically associated with pulmonary embolism in 99% of cases [1]. It is a very poorly described entity with a prevalence of 4% in the literature [2, 3]. Several parameters have been identified as predictors of this pathology. The factors generally associated with its occurrence are congestive heart failure, right bundle branch block, and right ventricular systolic dysfunction [4]. It is a serious disease with a mortality rate of between 28% and 44% [4]. Despite this high mortality rate, its management remains less well codified, as there are no recommendations to date [4]. Emergency thrombolysis seems to be the most recommended treatment, but the route of administration is not unanimously accepted by authors, although there are regional consensuses [5]. We conducted *a prospective cross‐sectional cohort study* to investigate the factors associated with right intracavitary thrombi in pulmonary embolism at the Bogodogo University Hospital.

## 2 Patients and Methods

### 2.1 Study Population

This was a descriptive and analytical cross‐sectional study in the Cardiology Department of the Bogodogo University Hospital during the period from March 05, 2017 to July 31, 2023. Patients admitted to the cardiology department with pulmonary embolism confirmed by CT scan of the pulmonary arteries were included in our study. In cases of hemodynamic instability requiring emergency thrombolysis, the diagnosis of PE was re‐established by thoracic CT scan after the emergency had been resolved.

### 2.2 Study Variables

The presence of right intracavitary thrombus was the study variable of interest. Independent variables included:

▪ Sociodemographic and clinical data including risk of hospital death.
▪ Electrocardiographic parameters including the presence of S1Q3T3, negative T waves from V1 to V3, right bundle branch block, right atrial hypertrophy.
▪ The Doppler echocardiographic parameters were: dilatation of the right cavities, presence of thrombus, and systolic function of the right ventricle assessed by Tricuspid Annular Plane Systolic Excursion (TAPSE).
▪ The CT scan parameters were: location of thrombus, uni‐ or bilateral character, and dilatation of the pulmonary artery.
▪ Evolutionary variables included mode of exit.

### 2.3 Ethical Considerations

Our study does not involve any risk for the participants. Participation in the study offers no financial remuneration and does not expose the patient to any additional risk other than that associated with his or her pathology. The study does not require the doctor to perform any additional act other than that which he or she undertakes for the patient concerned. The confidentiality of patients' personal information was respected during data processing. We have obtained the consent of the subjects concerned.

### 2.4 Statistical Analysis

All the data were entered on a microcomputer and analyzed using Epi‐Info software in its French version 7.2.5.0. The patients were subdivided into two groups according to whether echocardiography revealed a right intracavitary thrombus (Thrombus IC+) or not (Thrombus IC−). *χ* ^2^ and Fisher's exact tests were used in univariate analysis to determine the categorical variables associated with the occurrence of intracavitary thrombi. For quantitative variables, a Student's mean comparison test was used after verification of normality. In the absence of variable normality, the Kruskal–Wallis test was used to compare medians. The normality of the distribution of continuous variables was tested using graphical methods. A value of *p* < 0.05 defined the significance threshold for the association between the independent variables and the occurrence of pulmonary embolism. All variables with a univariate *p* < 0.2 were included in a multivariate logistic regression model to identify independent predictors of intracavitary thrombi.

## 3 Results

### 3.1 Clinical Characteristics of Patients

**Figure 1:**
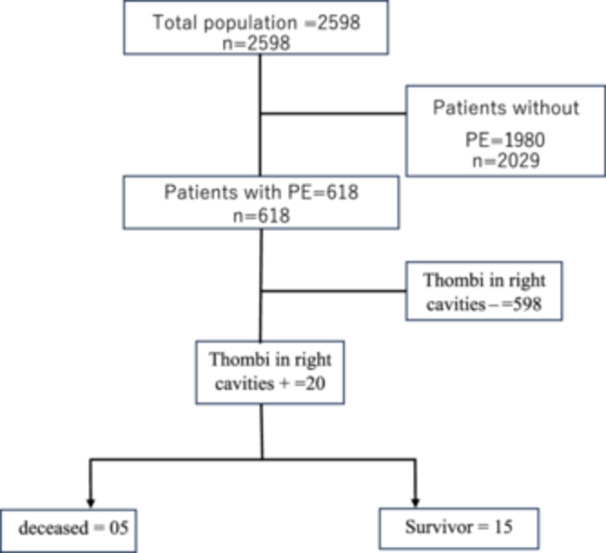
Patient flowchart.

Among the 618 patients hospitalized for pulmonary embolism, 20 cases of right intracavitary thrombi were recorded, representing a prevalence of 3.22%. Figure 1 shows the flow of our patients. The mean age of our series was 54 ± 16.74 years, with a sex ratio of 0.86 for women. A sedentary lifestyle and obesity were the predominant thromboembolic risk factors. Table 1 shows the general characteristics of the patients according to the presence or absence of thrombus in the right cavities.

**Table 1:** General characteristics of patients.

Variables	Total effectif (*n* = 618)	Thrombus+ (*n* = 20)	Thrombus− (*n* = 598)
Sociodemographic parameters			
Average age	54.46	53.11	54.56
Men	283	11	271
Women	335	09	326
Thromboembolic risk factors			
Obesity	171	04	167
Sedentary lifestyle	422	14	408
History of thromboembolic disease	64	01	63
Comorbidities			
Diabetes	45	00	45
Hypertension	161	05	156
Cancer	32	01	31
Chronic respiratory disease	38	03	35
Clinical signs			
Shortness of breath	515	16	499
Chest pain	491	16	475
Syncope/lipothymia	80	05	75
Haemoptysis	65	04	61
Arterial hypotension	62	06	56
Evolution			
Hospital death	62	05	57

### 3.2 Paraclinical Characteristics of Patients

The electrocardiogram showed an S1Q3T3 appearance in 05% (*n* = 07) of right intracavitary thrombi. The McGinn and White sign and the presence of a negative T wave from V1 to V3 were the most frequent electrical characteristics in our series. Transthoracic Doppler echocardiography revealed dilatation of the right cavities and right ventricular systolic dysfunction in 07.31% (*n* = 12) and 04.95% (*n* = 10) of these patients, respectively. Of the 20 cases of thrombi identified, 14 were in the right atrium, 5 in the right ventricle, and 1 in the pulmonary artery. PE was proximal in 11 patients and bilateral in 8 patients. Table 2 summarizes the paraclinical characteristics of patients.

**Table 2:** Electro–echocardiographic and scanographic characteristics of patients.

Paraclinical characteristics	Total number (*n* = 618)	TIC+ (*n* = 20)	TIC− (*n* = 598)
Electrocardiographic aspects			
Aspect S1Q3T3	140	07	133
Negative T waves from V1 to V3	102	02	100
Right bundle branch block	62	03	59
Right atrial hypertrophy	27	00	27
Echocardiographic findings			
Dilation of the right cavities	164	12	152
Right ventricular systolic dysfunction	202	10	192
TAPSE < 17 mmHg	109	09	100
CT scan			
Proximal pulmonary embolism	335	11	324
Bilatérale pulmonary embolism	346	08	338
Pulmonary artery dilatation	36	01	35

Abbreviation: TIC, intrcavitary thrombus.

### 3.3 Therapeutic and Evolutionary Characteristics of Patients

Heparin was used in all patients with right intracavitary thrombi. The presence of right intracavitary thrombi was statistically associated with thrombolysis. In total, 25% of patients with right intracavitary thrombi died. Table 3 gives the characteristics of the patients in univariate analysis. After multivariate logistic regression, arterial hypotension (OR = 4.14; IC = 1.53–11.2; *p* = 0.0051), right ventricular dilatation (OR = 4.40; IC = 1.76–10.9; *p* = 0.0051), thrombolysis (OR = 9.21; IC = 3.69–23.3; *p* = 0.00), and death (OR = 3.16; IC = 1.10–9.02; *p* = 0.03) were associated with the presence of intracavitary thrombi with a *p* < 0.05 (Table 4).

**Table 3:** Bivariate analysis of general patient characteristics.

Variables	TIC+ (*n* = 20)	TIC− (*n* = 598)	Odds ratio	*p*
Thromboembolic factors				
Sedentary lifestyle	14 (3.32%)	408 (96.68%)	1.0866	0.5416
Obesity	04 (2.34%)	167 (97.66%)	0.6452	0.3097
VTE history	1 (1.56%)	63 (98.44%)	0.4470	0.3674
Comorbidities				
Hypertension	05 (3.11%)	156 (96.89%)	0.9444	0.5745
Cancer	02 (6.25%)	30 (95.75%)	2.1037	0.2774
Respiratory disease	03 (7.89%)	35 (92.11%)	2.8387	0.1183
Clinical signs				
Shortness of breath	16 (3.11%)	499 (96.89%)	0.7936	0.4341
Chest pain	16 (3.26%)	475 (96.74%)	1.0358	0.6059
Syncope	05 (6.25%)	75 (93.757%)	2.3244	0.0685
Haemoptysis	4 (6.15%)	61 (93.85%)	2.2008	0.1489
Hypotension	06 (9.68%)	56 (90.32%)	4.1482	0.0100
Paraclinical signs				
S1Q3T3 Aspect	7 (5.00%)	133 (95.00%)	1.8826	0.1442
T wave V1 to V3	7 (07.44%)	87 (92.55%)	0.0358	0.4034
CR dilatation	12 (7.32%)	152 (92.68%)	4.4013	0.0013
RV dysfunction	10 (4.98%)	191 (95.02%)	2.1309	0.0759
Proximal PE	11 (3.18%)	335 (96.82%)	0.9595	0.5514
Treatment				
Thrombolysis	11 (13.58%)	70 (86.42%)	9.2190	0.0000
Heparin	20 (3.24%)	598 (96.76)	0.0000	0.0000
Evolution				
Deceased	05 (8.06%)	57 (91.94%)	3.1637	0.0409

Abbreviations: CR, right cavity; PE, pulmonary embolism; VR, right ventricular; VTE, venous thromboembolism.

**Table 4:** Multivariate analysis of variables associated with right intracavitary thrombi.

Variable	Univariate analysis		Multivariate analysis		
	OR	*p*	OR	[IC 95%]	*p*
Hemoptysis	2.2008	0.1489	2.2015	[0.71; 6.69]	0.1700
Chronic respiratory disease	2.8387	0.1183	0.0000	[0.00; 1.00]	0.9759
Arterial hypotension	4.1482	0.0100	4.1485	[1.53; 11.2]	0.0051
Aspect S1Q3T3	1.8826	0.1442	1.8826	[0.73; 4.81]	0.1866
Syncope	2.3244	0.0685	2.3253	[0.82; 6.58]	0.1119
Dilation of the right ventricle	4.4013	0.0013	4.4013	[1.76; 10.9]	0.0015
Thrombolysis	9.2190	0.0000	9.2191	[3.69; 23.3]	0.0000
Deaths	3.1637	0.0000	3.1638	[1.10; 9.02]	0.0313

*Note:* After multivariate analysis using logistic regression, arterial hypotension, right ventricular dilatation, thrombolysis, and death were significantly associated with the presence of right heart thrombi with a *p* < 0.05.

## 4 Discussion

Thrombi of the right heart can be classified into two main groups: the first group represents thombi of venous origin in transit from the right atrium or right ventricle. These are generally mobile, serpiginous thrombi [6]. Thrombi developed in situ or mural are small and immobile and make up the second group [4]. In the first group, thrombi most often originate in the veins of the lower limbs, inferior vena cava, renal veins, etc. The frequency of right heart thrombi in our series was 3.22%. Higher frequencies have been reported in the literature. The International Cooperative Pulmonary Embolism Registry (ICOPER) study found 4% [2]. Like other series, our results show a frequent association between hemodynamic instability and the presence of right heart thrombi in PE. The association of right ventricular dysfunction and arterial hypotension with right heart thrombi has been described in many series. It was 20% in the series by Koć et al. and that of Kurnicka et al. [7, 8].

The thromboembolic risk factors reported in our series were similar to those found in other scientific reviews. In agreement with previous studies, a sedentary lifestyle, obesity, and cancer were the most important thromboembolic risk factors associated with right heart thrombi [9]. Hypertension was the most frequent comorbidity in our series, but was not significantly associated with right heart thrombi. The difference between the physical characteristics of left and right heart thrombi is that right intracavity thrombi most often do not originate from the right atrium but rather from emboli of venous origin [10]. However, although exceptional, heart failure or atrial fibrillation may be responsible for right heart thrombi [10].

In our series, no thrombus attached to the heart wall was observed. That could be due to the size, location of thrombi in the heart chambers or the experience of the sonographer. Some authors also believe that the biological characteristics of thrombi may limit their detection by transthoracic echocardiography [11].

Emergency fibrinolysis was carried out in 13.58% of patients in our series, or 55% in the case of right intracavity thrombi, with a statistically significant difference. Failure to treat leads to mortality in 100% of cases [12]. However, there is still no consensus on the management of these thrombi, as there is as yet no consensus among practitioners. As a result, it is not clearly defined in the European recommendations on the management of pulmonary embolism [13]. Elsewhere, embolectomy has given satisfactory results [4, 14]. Some authors believe that thrombolytic agents cause thrombi to migrate into the pulmonary system, with dreadful consequences [15]. For this reason, surgical embolectomy is sometimes preferred to thrombolysis. In the case of early discovery of deep vein thrombosis in the lower limbs, the installation of a vena cava filter can prevent these thrombi from passing through the right cavities to cause PE [16].

The mortality rate was 25% among patients with right intracavitary thrombi in our series. This result is lower than that of Seghda and colleagues who found 45% mortality in 2015 [3]. Lower values have been reported in the literature. Indeed, Islam and colleagues, Ibrahim and colleagues, and Athappan and colleagues reported, respectively, 18.7%, 20.4%, and 23.2% mortality of right heart thrombi [16, 17, 18, 19]. The absence of emergency treatment other than fibrinolysis could explain this high mortality in our context.

## 5 Limitations of the Study

The limitations of this study are its single‐center design, the no 30‐day follow‐up, and the absence of investigations with an etiological aim. These limitations make it impossible to generalize these results, hence, the need for further multicenter studies.

## 6 Conclusion

The presence of a floating thrombus in the right chambers is an uncommon entity but is associated with a high mortality rate. Arterial hypotension, right ventricular dilatation, thrombolysis, and death were the factors associated with right heart thrombi in this study. Although their mortality is high, there is no consensus or recommendation on their management. Surgical embolectomy or emergency fibrinolysis appear to be the best way of improving their prognosis. An evaluation of the impact of thrombolysis on right intracavitary thrombi is crucial in our context where other means are unavailable.

## Author Contributions

**Wendlassida Martin Nacanabo:** writing – original draft, writing – review and editing, investigation, methodology. **Taryètba André Arthur Seghda:** conceptualization, methodology, data curation, writing – original draft, writing – review and editing, investigation, project administration. **Djième Claudine Dah:** methodology, data curation. **Mureille Loya:** data curation. **Lamoudi Prisca Thiombiano:** data curation. **Wendlassida Léa Françoise Sawadogo:** data curation. **Ella Hatoula Lengani:** data curation. **Anna Tall/Thiam:** supervision. **Nobila Valentin Yameogo:** supervision. **André Koudnoaga Samadoulougou:** supervision. **Partice Zabsonre:** supervision.

## Ethics Statement

Approval was obtained from the Ethics Committee of Bogodogo University Hospital (N2024‐03‐038). The procedures used in this study adhere to the tenets of the Declaration of Helsinki.

## Conflicts of Interest

The authors declare no conflicts of interest.

## Transparency Statement

The lead author Wendlassida Martin Nacanabo affirms that this manuscript is an honest, accurate, and transparent account of the study being reported; that no important aspects of the study have been omitted; and that any discrepancies from the study as planned (and, if relevant, registered) have been explained.

## Data Availability

The data that support the findings of this study are available on request from the corresponding author. The data are not publicly available due to privacy or ethical restrictions.
